# Therapeutic potential of exosomes/miRNAs in polycystic ovary syndrome induced by the alteration of circadian rhythms

**DOI:** 10.3389/fendo.2022.918805

**Published:** 2022-11-16

**Authors:** Wei-hong Chen, Qiao-yi Huang, Zhi-yi Wang, Xuan-xuan Zhuang, Shu Lin, Qi-yang Shi

**Affiliations:** ^1^ Department of Gynaecology and Obstetrics, the Second Affiliated Hospital of Fujian Medical University, Quanzhou, Fujian, China; ^2^ Centre of Neurological and Metabolic Research, the Second Affiliated Hospital of Fujian Medical University, Quanzhou, Fujian, China; ^3^ Group of Neuroendocrinology, Garvan Institute of Medical Research, Sydney, NSW, Australia

**Keywords:** Polycystic ovary syndrome, miRNAs, circadian rhythm, exosomes, inflammation, oxidative stress

## Abstract

Polycystic ovary syndrome (PCOS) is a reproductive dysfunction associated with endocrine disorders and is most common in women of reproductive age. Clinical and/or biochemical manifestations include hyperandrogenism, persistent anovulation, polycystic ovary, insulin resistance, and obesity. Presently, the aetiology and pathogenesis of PCOS remain unclear. In recent years, the role of circadian rhythm changes in PCOS has garnered considerable attention. Changes in circadian rhythm can trigger PCOS through mechanisms such as oxidative stress and inflammation; however, the specific mechanisms are unclear. Exosomes are vesicles with sizes ranging from 30–120nm that mediate intercellular communication by transporting microRNAs (miRNAs), proteins, mRNAs, DNA, or lipids to target cells and are widely involved in the regulation of various physiological and pathological processes. Circadian rhythm can alter circulating exosomes, leading to a series of related changes and physiological dysfunctions. Therefore, we speculate that circadian rhythm-induced changes in circulating exosomes may be involved in PCOS pathogenesis. In this review, we summarize the possible roles of exosomes and their derived microRNAs in the occurrence and development of PCOS and discuss their possible mechanisms, providing insights into the potential role of exosomes for PCOS treatment.

## 1 Introduction

Polycystic ovary syndrome (PCOS) is a common gynecological endocrine disorder that affects up to 18% of women (1). Patients with PCOS experience multiple severe clinical sequelae, including reproductive complication (menstrual disorders, infertility, and hyperandrogenism) (2, 3), metabolic dysfunction (insulin resistance [IR] and diabetes) (4), and psychological disorders (depression, anxiety disorder, social phobia) (5), which negatively impact quality of life of patients. Given the heterogeneity and clinical characteristics of PCOS, symptoms may manifest differently among patients (3). Researchers have shown that endocrine and metabolic abnormalities in women with PCOS may be associated with the severity of hyperandrogenism (6). Treatments, which include lifestyle changes, drug therapy, and surgery, are typically focused on symptom relief and do not yield satisfactory results. Although PCOS has been recognized for decades, its pathophysiological mechanisms remain unclear. The circadian rhythm(CR) affects reproduction by regulating various functions of the hypothalamic-pituitary-gonadal (HPG) axis and the ovaries (7). Dysfunction of the HPG axis is critical in the development of PCOS (8, 9). Additionally, CR disorders may affect reproductive outcomes by inducing IR, oxidative stress (OS), and systemic inflammation (10). Wang et al. found that night shift work (for >2 years) remarkably correlates with PCOS risk (11). A recent study suggests that circadian rhythm disorders may be one of the causes of excess androgen in PCOS (12). Therefore, alterations in circadian rhythm may be associated with PCOS.

Exosomes, small extracellular vesicles (EVs), typically 30–120 nm in diameter, widely participate in the regulation of various physiological processes and play an important role in PCOS pathogenesis. Exosomes carry unique macromolecules, including proteins, lipids, DNA, mRNA, and non-coding RNA (ncRNA), and deliver genetic information to recipient cells, acting as “bridges” in cellular communication and affecting the functions of target cells (13, 14). In this review, we (1) discuss the role and possible mechanisms of exosome-mediated regulation of CR change-induced PCOS, (2) address the relationship between CR and PCOS, and (3) explore novel insights into the application of exosomes for treating PCOS.

## 2 CR regulation of metabolism and fertility

### 2.1 The circadian clock network

**Figure 1:**
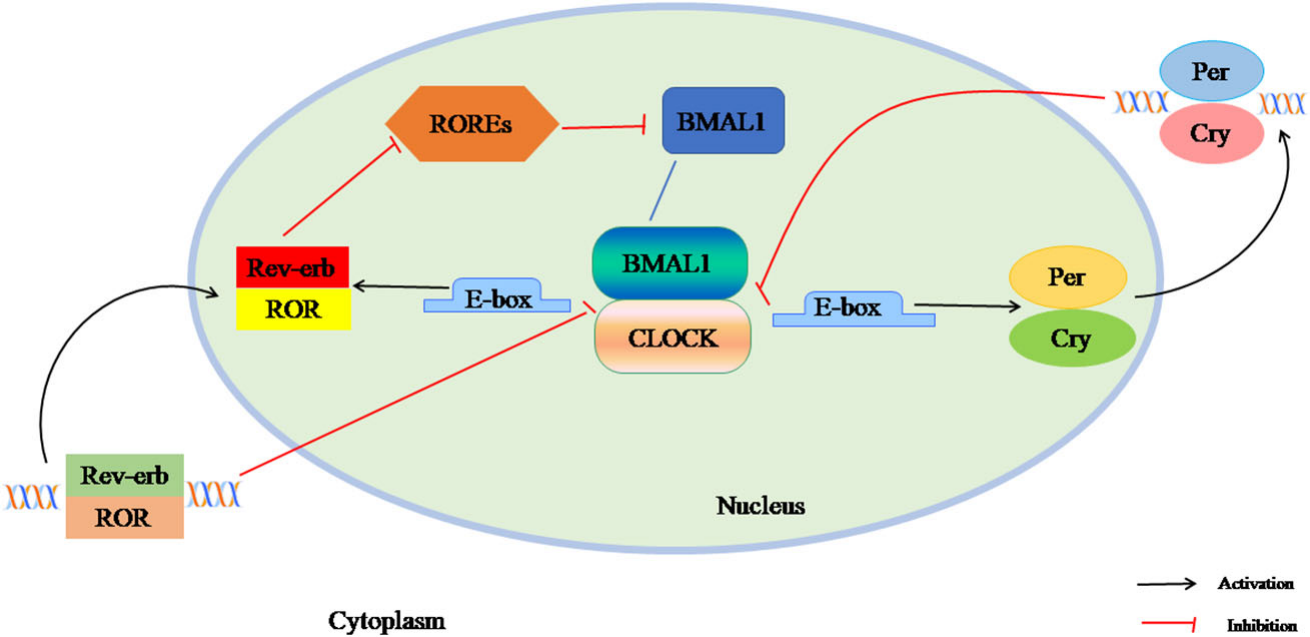
The composition of circadian clock. The mammalian circadian clock network is driven by several transcription factors that control the core feedback loop, including the transcription activators CLOCK, BAML1, and the clock genes Per1, Per2, Per3and Cry1, Cry2. In the first circuit, the BMAL1 and CLOCK heterodimer binds to E-box and E-box-like promoter elements, activating the transcription of Per and Cry genes. As transcription increases, PER and CRY accumulate in the cytoplasm and their dimers enter the nucleus, inhibiting CLOCK: BMAL1 activity and stopping gene expression. The second feedback loop is composed of nuclear receptor Rev-erbα / β and Rorα-γ. Rev-erbs and RORs competitively bind to ROREs to regulate BMAL1 transcription and maintain a stable circadian clock cycle.

The mammalian circadian clock network is driven by several transcription factors that control the core feedback loop, including the transcription activator circadian locomotor output cycle kaput (CLOCK), brain and muscle Arnt-like protein 1 (BAML1), and the clock genes Period *(Per1*, *Per2*, *Per3)* and Cryptochrome *(Cry1*, *Cry2).* In the first circuit, the BMAL1 and CLOCK heterodimer bind to E-box and E-box-like promoter elements, activate the transcription of *Per* and *Cry* genes. As transcription increases, PER and CRY accumulate in the cytoplasm and their dimers enter the nucleus, inhibiting CLOCK: BMAL1 activity and stopping gene expression. The second feedback loop is composed of nuclear receptors Rev-erbα/βand Rorα-γ. Rev-erbs and RORs competitively bind to ROR response elements (ROREs) to regulate BMAL1 transcription and maintain a stable circadian clock cycle (**Figure 1**). The CLOCK : BMAL1 heterodimer also drives the expression of thousands of clock-regulated genes controlling many biological processes such as metabolism (15–17).

### 2.2 CR regulation of metabolism

#### 2.2.1 BMAL1 and metabolic regulation

Knockdown of *BMAL1* suppresses metabolic rhythm, whereas interference with *Cry1* or *Cry2* typically shortens or prolongs metabolic rhythm, respectively (18). *BMAL1* plays an important role in lipid and glucose metabolism. Zhang et al. found that *BMAL1* promotes adipogenesis through the insulin - mTOR complex 2 (mTORC2) – protein kinase B (Akt) signaling pathway in the liver tissue (19). In addition, in specific *BMAL1* knockout (KO) mice, the CR of insulin sensitivity is impaired and IR is induced (20). During fasting, glucagon induces the transcription of *BMAL1* by activating cyclic adenosine response element-binding protein 1(CREB)/CREB-regulated transcription coactivator 2 (CRTC2), while insulin inhibits *BMAL1* expression by suppressing the activity of CREB/CRTC2 (21). In another study, the regulatory effect of the molecular clock on white adipose tissue physiology was observed in CLOCK-KO mice. KO of *Per2* or RORα/γ was beneficial for adipogenesis, whereas deletion of *BMAL1* or Rev-erbα suppressed adipogenesis (22).

#### 2.2.2 SIRT1 pathway

Both the deacetylase Sirtuin1 (SIRT1) and AMP-activated protein kinase(AMPK) are sensitive to energy metabolism and therefore have synergistic effects (23). SIRT1 is involved in the circadian transcription of core clock genes such as *BMAL1*, *Per2* and *Cry1*. SIRT1 binds to CLOCK : BMAL1 through circadian rhythm (24). Nicotinamide phosphoribosyltransferase (NAMPT) and nicotinamide adenine dinucleotide(NAD)^+^ show circadian oscillation patterns and NAMPT participates in IR by regulating peroxisome proliferator-activated receptor γ (PPARγ) and adiponectin through SIRT1 (25). A reduction in NAMPT-mediated NAD^+^ biosynthesis stimulates oscillation of the clock gene *Per2* by releasing SIRT1-mediated CLOCK : BMAL1. In turn, CLOCK binds and upregulates NAMPT to form a feedback loop of NNMPT/NAD^+^ and SIRT1/CLOCK : BMAL1 (26). Zhai et al. showed decreased expression of *BMAL1* in the liver and adiposetissue of PCOS rats, resulting in suppression of the NAMPT/NAD^+^/SIRT1 pathway and the promoting on of IR (27). We speculate that SIRT1 may act as a bridge between *BMAL1* and IR disorders in patients with PCOS. A recent study reported that activation of SIRT1 in diabetic rats can significantly up-regulate the expression of *BMAL1* and increase the activation of autophagy to alleviate myocardial ischemia/reperfusion injury (28). This provides new insights into the treatment of PCOS, but more research is needed to validate these findings.

### 2.3 CR regulation of female fertility

Recent studies have confirmed that biological rhythm disorders lead not only to metabolic dysfunction in PCOS, but also cause reproductive disorders (29). In the hypothalamus, the suprachiasmatic nucleus (SCN) produces timing signals to activate gonadotropin-releasing hormone (GnRH) neurons and stimulates pituitary gonadotropin cells to release luteinizing hormone(LH) (30). Bahougne et al. showed that exposure to anacutetime displacement in female mice can lead to moderate and temporary changes in LH surge, while exposure to chronic displacement can lead to severe, rapid and lasting changes in LH surge before ovulation, resulting in decreased fertility (31). In humans, disruption of circadian rhythms negatively affects fertility. A cohort study of women in North America found that shift workers had lower fertility rates than daytime workers (32). A study also confirmed an increase in LH concentrations during night/shift work (33). And another cross-sectional study reported that sleep disorders are twice as common in PCOS women compared to non-PCOS women (34).

In addition, clock genes play an important role in female fertility. Clock gene expression rhythms have been reported in the HPG axis of mice, rats, and humans (35). For example, in rats, *BMAL1* mRNA expression is highest in the presence of light and Per2 mRNA expression is highest when in the absence of light (36). Long-term light exposure desynchronizes the clock genes (*BMAL1, clock, Per1, Per2, Cry1 and Cry2*) in the central (hypothalamus containing SCN) and peripheral (ovary and uterus) organs of hamsters, thus affecting ovarian hormone regulation, embryo implantation and pregnancy success rates (37). A BMAL1 KO can cause reproductive disorders in mice. In addition, Per1 / Per2 double mutations mice have reduced ovarian follicular reserves, leading to a decline in fertility. (38, 39) Studies have shown that BMAL1-KO mice exhibit abnormal follicle development, reduced fertilization rates, delays in early embryo/blastocyst developmental (40, 41). Chen and colleagues found that down-regulation of *BMAL1* expression inhibits the synthesis of progesterone and prostaglandin E2, which are key players in the reproductive process (42). *Per1* and *Per2* KO mice are characterized by a significant decrease in fertility due to irregular oestrous cycles (43). Furthermore, middle-aged *Cry* gene-deficient female mice show early estrus abnormalities leading to reduced fertility, which can be alleviated by adjusting the light/dark cycle (44). Hence, reproductive pathophysiological treatment strategies based on the CR system is another unexplored field with a great application potential.

**Figure 2:**
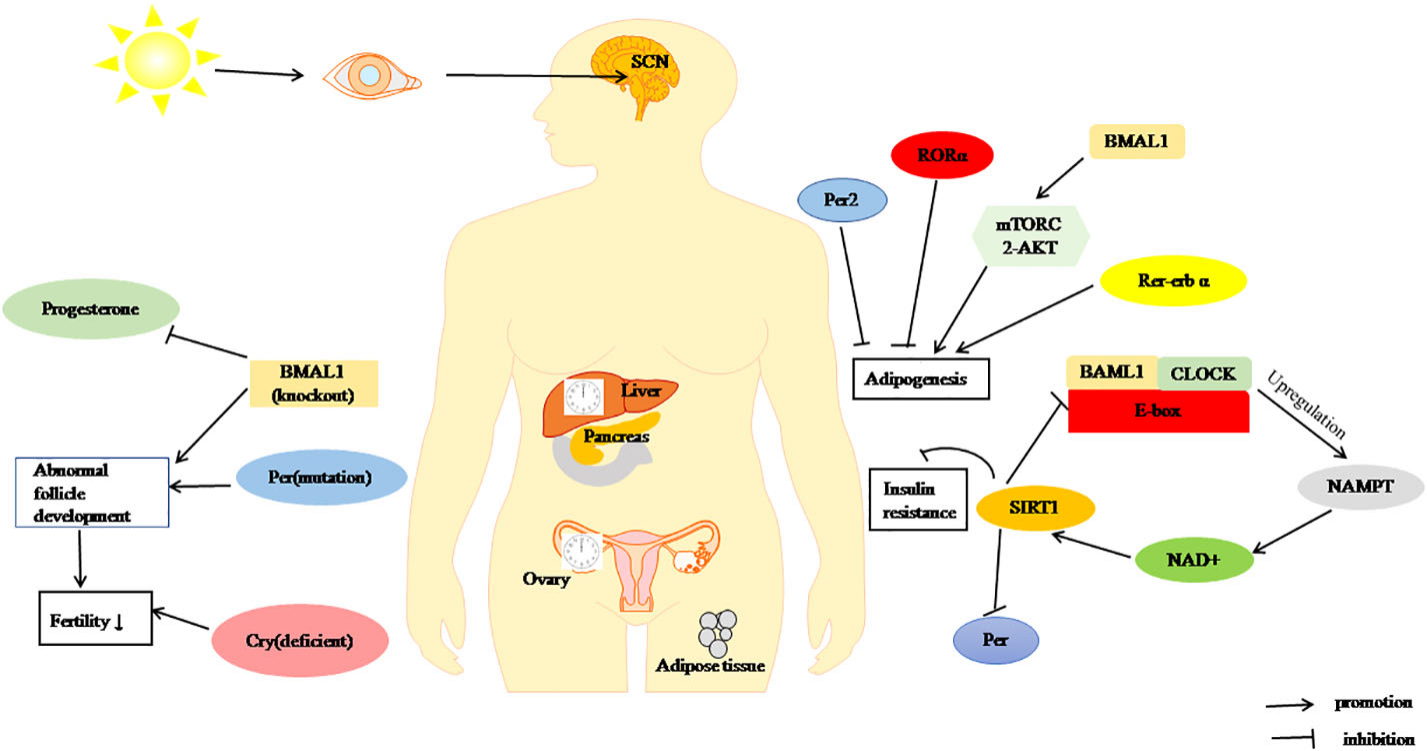
Regulation of metabolism and fertility by the CR. BMAL1, CLOCK and Per through SIRT1 pathway inhibit of insulin resistance. RORα, Per2, and Rer-erbα can inhibit adipogenesis, BAML1 promote adipogenesis by mTORC2/Akt signalling pathway. BMAL1-KO, Cry deficient, and Per1/2 mutation cause decreased fertility.

In short, alterations in CR may be involved in the progression of PCOS and regulate its reproductive and metabolic processes through its own genes (**Figure 2**), which provides a new strategy for the treatment of PCOS.

## 3 Mechanism underlying the correlation between circadian rhythm, oxidative stress and inflammation

Chronic inflammation and OS are involved in the pathogenesis of PCOS. Abnormal expression of pro-inflammatory cytokines, characterized by increased levels of C-reactive protein (CRP), serum interleukin 1 (IL-1), interleukin 6 (IL-6), interleukin 8 (IL-8) and chemokine C-C motif ligand 2 (CCL2) has been implicated in the aetiology of PCOS (45, 46). Oxidative stress is closely associated with inflammation. Inflammation induces ROS production, and oxidative stress exacerbates inflammation (47). Clinical manifestations of PCOS may result in the development of the local and systemic OS, which in turn induces a proinflammatory state that promotes IR and hyperandrogenemia in PCOS (48). Levels of some circulating antioxidant biomarkers are decrease in patients with PCOS, including those of Paraoxonase-1(PON1) (49). However, the activity of other antioxidant biomarkers, such as catalase (CAT) and superoxide dismutase (SOD), is significantly elevated in some patients (50).

Circadian rhythm disorders may lead to oxidative stress and systemic inflammation (10).

### 3.1 CR changes exacerbate OS in women with PCOS

OS is a state of imbalance between oxidation and anti-oxidation in the body. In patients with PCOS, IR is closely related to inflammation and increased OS (23, 51). The circadian clock is the major regulator of ROS homeostasis. Animals with mutated circadian protein showed elevated ROS levels and oxidative damage (52, 53). Tomas et al. demonstrated that continuous light attenuates or disrupts the CRs of the antioxidants SOD and CAT in Syrian hamsters (54).

The Kelch-like ECH-associated protein 1 (Keap1)/nuclear factor-erythroid 2-related factor 2(Nrf2) pathway proved to be a critical pathway in OS regulation (55). Studies have shown that Nrf 2 is a transcriptional regulatory target of *BMAL1*. *BMAL1* not only activates the Nrf2-mediated antioxidant pathway, but also affects the expression of Nrf2-mediated antioxidant genes such as heme oxygenase 1(*HO-1)* and NAD(P)H dehydrogenase (quinone) 1(*NQO-1)* (56). In addition, BMAL1 regulates homeostasis by directly regulating ROS levels. For example, specific KO of *BMAL1* in mouse pancreatic β cells restrained glucose-activated insulin secretion (GSIS) and elevate ROS sensitivity, resulting ROS overproduction (57). Background of BMAL1 in the peripheral tissue of PCOS, we surmise that the Nrf2 pathway can regulate *BMAL1* to improve OS, which provides a new perspective for the treatment of PCOS.

### 3.2 CR changes exacerbate inflammatory responses in women with PCOS

The circadian clock has been suggested to play a key role in the rhythmic regulation of inflammatory responses (58). Clock genes regulate the expression of various inflammatory cytokines. *Cry *impacts IL-1β, IL-6 and tumor necrosis factorα (TNFα) (59), *RORα* affects IL-1β and IL-6 (59), *Per2* regulates IFNγ and IL-1β (60); *Per1* is related to monocyte chemotactic protein-1(MCP-1) (61); *Rev-erbα*modulates IL-6 (62) and TH17 cells (63). Secretion of TNFα and IL-6 shows circadian oscillations. KO of *Rev-erb*α leads to impaired circadian regulation of inflammatory responses. Furthermore, deletion of *BMAL1* in macrophages down-regulates Nrf2 induction, leading to reduced antioxidant responses and increased IL-1β production (57, 64). As reported that the shift-workers, who often suffer from circadian rhythm disorders, may resulting PCOS, and the level of inflammatory markers present increased (65).We hypothesized that PCOS could be improved by modulating clock genes associated with OS and inflammation; however, more studies are needed to confirm this hypothesis.

### 3.3 Changes in PCOS-related hormone levels caused by continuous light exposure

Studies have shown that light stimulates LH secretion (66). The possible mechanisms include inhibition of melatonin (MT) secretion, projection of SCN to the hypothalamic-pituitary-ovarian (HPO) axis, and abnormal expression of circadian genes (67). Prolonged exposure to light induces changes in the ovarian morphology and hyperandrogenemia in rodents, suggesting that CR changes may trigger PCOS (68, 69). In addition, studies have shown that continuous exposure of light in humans and animals to light also leads to increased levels of follicle-stimulating hormone (FSH) and estradiol (E2) (70, 71).However, another study showed that persistent darkness leads to increased serum LH/FSH ratio and testosterone levels in rats (29).

Melatonin levels are regulated by the photoperiod, which increases its production and secretion at night, in response to darkness, inhibits its secretion in response to light (72). Zhang and colleagues demonstrated that continuous exposure to light significantly reduces the level of MT in mice, which in turn reduces the release of LH, oestrogen, androgen, and progesterone by inhibiting the hypothalamic-pituitary-ovarian(HPO) axis, suggesting that a reduction of MT could be the underlying mechanism of hyperandrogenemia (66). MT can also increase FSH secretion by stimulating the pituitary gland (73). Tagliaferri et al. found a significant increase in FSH levels, significant recovery in menstrual cyclicity, and improvement androgen balance in women with PCOS, following a 6-month treatment with oral melatonin (74). Another research reported that administered 12 weeks of melatonin supplementation, hirsutism improved significantly and testosterone levels decreased significantly (75). Moreover, Li et al. verified that melatonin attenuates persistent darkness-induced hyperinsulinemia and hyperandrogenism in PCOS rats *via BMAL1, Per1 and Per2* (29).

Take together, targeting melatonin expression by altering circadian regulation is a potential therapeutic strategy for PCOS; however, the relevant evidence is still insufficient, and more in-depth studies are needed to verify this hypothesis and explore the precise underlying mechanisms.

## 4 Exosome and PCOS

### 4.1 Origin and secretion regulation of exosomes

#### 4.1.1 Origin of exosomes

Extracellular vesicles are important intercellular messengers for proteins and ncRNA and can be divided into exosomes, microvesicles and apoptotic bodies according to their size, content, biogenesis and specific surface markers. Exosomes are lipid bilayer vesiclessecreted from the extracellular space in response to specific stimuli under physiological or pathological conditions. Exosomes transfer specific molecular cargoes, particularly miRNAs (76, 77). MiRNAs are small 20–23 nucleotide ncRNA molecules that negatively regulate gene expression via mRNA cleavage or translation. Abnormal expression of miRNAs is implicated in the pathogenesis of many conditions, such as obesity and diabetes, as well as in sex hormone synthesis. In recent years, the role of miRNAs in PCOS pathology has garnered considerable attention (78, 79) (**Table 1**).

**Table 1:** Expression and regulation of miRNAs in the target genes/pathways related to PCOS and the circadian rhythm.

miRNA	Target gene/pathway	Express level(↑/↓)	References
miR-93	CDKN1A, GLUT4	↑	(80–82)
miR-21	LATS1	↑	(82)
miR-27b	PPAR-γ	↑	(83)
miR-103	IRS1, P13K/AKT	↑	(84, 85)
miR-155	BMAL1、PDCD4	↑	(86, 87)
miR-320	P13K, GLUT4, E2F1, SF-1	↓(in serum),↑(in granulosa cells)	(81)
miR-135	IL-8	↑	(81)
miR-146a	TNF-α, IL-6, IRAK1, IL-1β	↑	(77, 81, 88)
miR-9	IL-8	↑	(81)
miR-132	HMGA2	↓	(81)
miR-18b	IL-8	↑	(81)
miR-23a	–	↓	(89)
miR-24	CYP11A1	↓	(90)
miR-19	–	↓	(91)
miR-200b	PTEN	↑	(81)
miR-483-5p	P13K/Akt	↓	(81)
4miR-24-3p	Per2, CYP11A1	↓	(92, 93)
miR-151-3p	–	↓	(93)
miR-34a	Per1	–	(86)
miR-194	Pers	↑	(80, 94)
miR-193b	–	↑	(80)
miR-122	PPARγ	↑	(80, 95)
miR-33b-5p	GLUT4	↑	(80)
miR-233	GLUT4, Keap1-Nrf2	↑	(80, 96)
miR-133a/b	GLUT4	–	(97)
miR-223	GLUT4, Keap1-Nrf2	–	(97, 98)
miR-143	GLUT4	–	(97)
miR-199a-3p	–	↑	(76)
miR-199a-5p	–	↑	(76)
miR-23a/b	–	↓	(89)
miR-30c	–	↑	(81)
miR-33	ABCA1	–	(99)
miR-185	Cry1	–	(100)
miR-148a	LDLR, ABCA1	–	(100)
miR-375	PPAR-γ	–	(101)
miR-219	CLOCK/BMAL1, PER1	–	(86, 94)
miR-494	BMAL1	–	(86, 94, 102)
miR-27b-3p	BMAL1	–	(102)
miR-142-3p	BMAL1	–	(102)
miR-433	Per2, BMAL1	–	(86)
miR-96	Per2	–	(103)
miR-30a-5p	Per2	–	(92)
miR-25-3p	Per2	–	(92)
miR-181a	Per3	–	(104)
miR-455-5p	Clock mRNA	–	(105)
miR-126	P13K	↑	(81, 97)
miR-384-5p	P13K	↑	(97)
miR-29	P13K	↑	(97)
miR-1	P13K	↑	(97)
miR-19a	P13K	–	(81)
miR-20b-5p	Akt	–	(77)
miR-141	Keap1	–	(106)
miR-28	Nrf2	↓	(107)
miR-153	Nrf2	↓	(107)
miR-708	Nrf2	↓	(107)
miR106b-5p	MAPK	↓	(108)
miR-141-3p	MAPK	↓	(108)
miR-221-3p	MAPK	↓	(108)
miR-374a-5p	IL-17A, CCL2	–	(109)

“-” refers to not mentioned or not found in the article. “↑” refers to increased or upregulated. “↓” refers to decreased or downregulated.

#### 4.1.2 Regulation of exosome secretion

Exosomes can be produced and released by different subtypes of endosomes through different mechanisms and function as cell types and physiological states. Exosome secretion can be increased or decreased under pathological conditions. OS promotes exosome release during endoplasmic reticulum stress (110). Exosome secretion is also mediated by various physical, chemical, and biological stimuli such as ultrasound (111), ionizing radiation (112), DNA damage (113), enzymatic influence (114), and inflammatory stimulation (115). Therefore, interfering with exosome release and damaging exosome-mediated intercellular communication are potential therapeutic strategies.

### 4.2 Role of exosomes in PCOS

#### 4.2.1 Effect of miRNAs on hyperandrogenism

In recent decades, more and more researchers have paid attention to the relationship between miRNA and abnormal androgen secretion in PCOS. Androgen affects follicular growth and health, and functions mainly through the androgen receptor (AR). AR levels are increased in patients with PCOS (79). Previous studies have confirmed that miR-21 and miR-93 are androgen response factors, which may be associated with follicular dysfunction in the pathogenesis of PCOS. Additionally, in patients with PCOS, miR-29a, miR27b, miR-103, miR-518, miR-320, and miR-155 are positively correlated with serum or free testosterone levels. In contrast, miR151 is negatively correlated with serum testosterone levels; the down-regulation of miR-23a in PCOS serum is also negatively correlated with testosterone levels. MiR-9, miR-18b, miR-132, miR-135 and miR-146a inhibit testosterone secretion. MiR-24 and miR-19 potentially reduce testosterone release in the medium of cultured human ovarian cells (79, 80, 91). The downstream target of AR is miR-200b, which is required for HPO axis-mediated ovulation. MiR-29c acts through the downstream pathways that affect androgen receptor localization. These results indicate that miR-200b and miR-29c are also closely related to hyperandrogenism in PCOS (116). Understanding the mechanisms by which miRNAs regulate androgen production can greatly contribute to improving the clinical symptoms and prognosis of PCOS. However, in-depth studies on how miRNAs cause androgen metabolism disorders in PCOS patients are currently lacking.

#### 4.2.2 Effect of miRNAs on IR inPCOS

IR is a common feature of PCOS. According to the statistics, about 50-70% of PCOS patients have IR and are at high risk for developing metabolic syndrome, type 2 diabetes mellitus, and inflammatory and cardiovascular diseases (51, 117). Studies have revealed that the levels of miR-194, miR-193b, and miR-122 are elevated in patients with PCOS, especially in those with impaired glucose metabolism. Overexpression of miR33b-5p, miR-93, and miR-233 plays an important role in IR by inhibiting glucose transporter 4 (GLUT4) expressions in patients with PCOS. In addition, overexpression of miR-223 increased the protein expression of GLUT4; however, it should be noted that miR-223 expression was increased only in women with PCOS who had IR. MiR-133a and miR-133b participate in the expression of the GLUT4 protein through the Kruppel-like transcription factor 15 (KLF15) and reduce insulin-stimulated glucose utilization to control IR, whereas, miR-143 is involved in GLUT4 expression (80, 97), GULT4 may be a target of miRNAs for IR treatment in PCOS patients. Moreover, the level of miR-146a was negatively correlated with IR and inflammatory factors (TNF-α and IL-6). However, Jiang et al. found no correlation between the expression of miR-146a in the exosomes of patients with PCOS and their glucose metabolism indicators (77, 118). Decreased expression of miR-24 in PCOS is associated with IR and abnormal PCOS-related hormones (90). In general, miRNAs play an important role in regulating glucose metabolism and IR pathogenesis in women with PCOS, and may be a potential target for treatment of IR-related PCOS symptoms.

#### 4.2.3 miRNAs affect obesity and lipid metabolism in PCOS

Obesity and dyslipidaemia are also common manifestations of PCOS. About 50 % of women with PCOS suffer from overweight or obesity (119). The expression levels of miR-21, miR-27b, miR-103, and miR-155 in women with PCOS and obesity are remarkably higher than in women with PCOS and normal weight (83). Xiong et al. found serum miR-23a/b expression was decreased in patients with PCOS, and that an increased body mass index (BMI) elevates serum miR-23b level, while miR-23a was not affected by BMI (89). MiR-30c regulates cholesterol biosynthesis and very low-density lipoprotein cholesterol (VLDL-C) secretion by reducing apolipoprotein production and becoming a target for treating hyperlipidaemia (120). Studies have shown that miR-122-5p and miR-223-3p are directly correlated with BMI, in contrast, miR-151a-3p/5p and miR-199a-3p/5p are negatively correlated with the BMI and waist-to-hip ratio (WHR) (121). MiR-122 regulates plasma low-density lipoprotein cholesterol (LDL-C) levels by inhibiting the secretion of VLDL (122). MiR-33 and lipid metabolism have been widely studied. The expression of miR-33 regulates ATP-binding cassette transporter A1 (ABCA1) and ATP-binding cassette subfamily G member 1 (ABCG1), and inhibition of miR-33 increases the expression of these proteins in the liver and elevates high-density lipoprotein levels. In animal models (mouse and rabbit), inhibition of miR-33 has been shown to modify the biosynthesis of VLDL-C and triglycerides. In addition, miR-33 affects cholesterol outflow and bile acid synthesis and excretion (99, 123–126). Furthermore, some miRNAs can regulate LDL-C metabolism. By inhibiting the expression of miR-148a, miR-128-1, and miR-185, circulating LDL-C levels were reduced (100). MiR-143 is up-regulated in the liver of obese mice, which inhibits insulin-stimulated Akt activity and glucose imbalance (127). In contrast, miR-375 expression promotes adipocyte differentiation by mediating PPARγ and extracellular signal-regulated kinases (ERK) activity (128). These findings suggest that miRNAs are closely associated with obesity and dyslipidaemia, and shed light on their potential as therapeutic targets for PCOS metabolism.

In summary, the relationship between miRNAs and PCOS progression is not fully understood, and the specific roles of miRNAs in PCOS development are unclear because one miRNA may have multiple mRNA targets and one mRNA may be controlled by multiple miRNAs. Therefore, further functional studies of miRNA-PCOS are needed.

## 5 miRNAs and Circadian rhythm

### 5.1 Regulation of CR by miRNAs

Increasing evidence has shown that miRNAs play important roles in maintaining the homeostasis of the circadian system. Cheng et al. found that miR-132 and miR-219 are involved in regulating CRs in mammals. Brain-specific miR-219 displays rhythmic oscillations in the SCN and targets the CLOCK : BMAL1 complex involved in cycle determination, whereas the light-activated expression of miR-132 needs CREB and mitogen-activated protein kinase (MAPK)/ERK. In addition, miR-494, miR-27b-3p, miR-155 and miR-142-3p are involved in the post-transcriptional regulation of clock gene *BMAL1* in the circulation. Rhythmic expression of miR-142-3p was observed in mouse SCN cells, which may be driven by a typical E-box. Changes in the expression of miR-192 and miR-194 not only affect the rhythm oscillation of BMAL1 mRNA, but also inhibit the expression of *Per* gene.*Cry1* translation is regulated by miR-185 (86, 94, 102, 129). Another study showed that miR-959-964 exhibits significant circadian oscillation (95).

**Figure 3:**
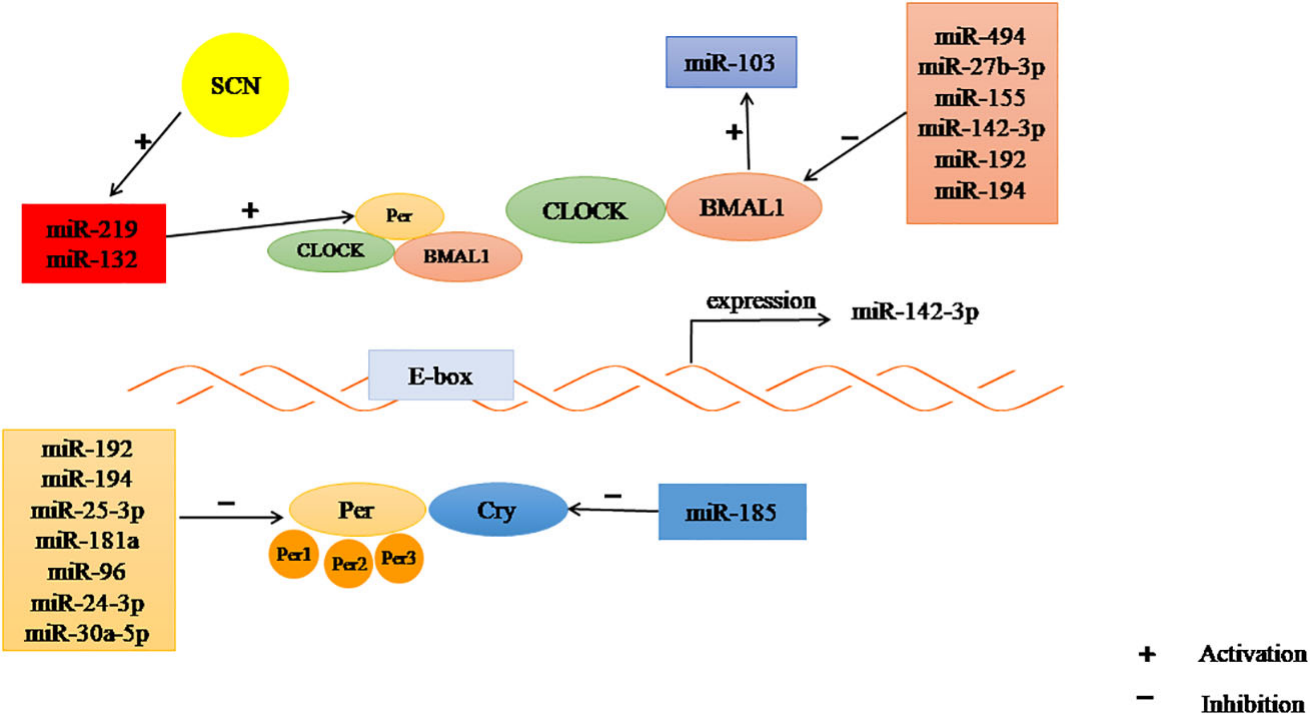
The regulation of CR by miRNAs. Some miRNAs may inhibit the expression of clock gene, some may promote.

Studies have shown that miR-96, miR-24-3p and miR-30a-5p directly target the core circadian clock gene *Per2*. MiR-25-3p was inversely expressed with the Per2 oscillation cycle. KO of miR-183/96/182 clusters in mice led to diurnal behavior changes (92, 103) Moreover, miR-181a directly targets *Per3* (104), and miR-455-5p regulates CRs by affecting the degradation of Clock mRNA (105). Yang et al. showed that circadian-regulated miRNAs in *Drosophila*, such as miR-263a and-263b, displayed robust, and moderate daily alteration (130). Xiao Chen et al. demonstrated that light-controlled miR-276a regulates CRs that influence *Drosophila* behavior by inhibiting the timeless(TIM) clock gene (131). In addition, the expression of miRNAs differs in different species. In humans, miR-107 regulates cell circadian oscillations by binding to clock genes. In mice, miR-17-5p is an important factor involved in circadian rhythm control (102) (**Figure 3**). Hence, the circadian rhythm can be changed by targeting miRNAs, to explore its mechanism and potential for treatment of PCOS.

### 5.2 Correlation between CR and miRNAs

Although miRNA-mediated post-transcriptional regulation regulates circadian oscillations, the circadian system in turn drives many miRNA expression-related CRs. Circadian regulation of RNA processing involves many steps, including mRNA capping, alternative splicing and tail length-controlled RNA stability changes, thereby promoting circadian gene expression. RNA degradation may also follow circadian patterns through miRNA rhythmic participation (102). Chen et al. found that *BMAL1* can stimulate miR-103 expression and regulate the CR expression of the rat cerebral artery CaV1.2 channel (large or long-term voltage-dependent Ca2^+^ channel) (84).

Wang et al. verified that transcripts including pri-mir-122 and pri-mir-24 exhibit strong circadian expression and are regulated by circadian rhythms (132). In addition, researchers showed that after two days of dark adaptation, the expression of pre-miR-219-1 and pre-miR-132 did not change significantly in the SCN tissues of *crypotochrome (mCry)1/mCry2* double mutant mice, indicating that the rhythmic expression of these two miRNAs are depended on the molecular clock (133).

Overall, miRNAs and CRs can interact with each other, but its influence on the regulation of the biological clock is complex. This complexity is largely due to various post-transcriptional, and post-translational mechanisms, and poses a challenge in exploring the mechanism of action and therapeutic potential of miRNAs in PCOS induced by circadian changes and, should be further evaluated in in-depth studies.

## 6 Possible pathways of miRNA-mediated PCOS

### 6.1 PI3K/Akt signaling pathway

Phosphatidylinositol 3-kinase (PI3K) plays an important role in PCOS, mainly by affecting granulosa cells (GC) proliferation and apoptosis (134). Xie et al. found that MT regulates autophagy and apoptosis through a PI3K-Akt pathway in PCOS rats, thus improving ovarian dysfunction (135). Another study showed that the P85 subunit is a potential target of miR-320. MiR-320a may be an important factor regulating IR by reducing insulin sensitivity through the PI3K signaling pathway (136, 137). Many miRNAs, such as miR-126, miR-384-5p, miR-29, miR1, and miR-19a, are involved in regulating PI3K. In addition, miR-483-5p regulates the proliferation of PCOS granulosa cells by activating the PI3K/Akt pathway (81, 97). MiR-20b-5p regulates insulin-stimulated glucose metabolism through the Akt signaling pathway. In addition, miR-20b-5p may alter the Akt signal routing in skeletal muscle cells (138). miR-103 and miR-497 regulate the PI3K/Akt pathway by targeting insulin receptor substrate (IRS) 1 (85).

### 6.2 MAPK/Nrf2 pathway

The Nrf2 signaling pathway acts as a center for regulating the antioxidant defense system in response to OS. As reported earlier, the activation of the Nrf2 signaling pathway attenuates OS and apoptosis in PCOS rats. MiR-223 may be a target of the Keap1-Nrf2 system in OS regulation (96). MiR-223 stimulates the Nrf2 signaling pathway by inhibiting Keap1 and inducing antioxidant defense system (98). MiR-141 may down-regulate the expression of its target gene *Keap1*, stimulate an increase of Nrf2, reduce ROS production and improve OS (106). Omar and colleagues confirmed that H_2_O_2_ treatment of bovine GCs increased the expression of *Nrf2* and decreased the expression of miR-28, miR-153 and miR-708, while regulating the expression of miR-153, miR-28 and miR-708 alone led to a decrease in the expression of Nrf2 and its downstream antioxidant genes. These results indicate that miRNAs are involved in regulating Nrf2-mediated OS response (107). Additionally, ROS can induce the activation of the MAPK pathway, which is blocked by antioxidants. H_2_O_2_-induced increases in ROS may inhibit the expression of specific miRNAs (miR106b-5p, miR-141-3p, miR-221-3p), which are critical for the activation of the MAPK pathway (108).

### 6.3 NF-κB pathway

NF-κB is a transcription factor involved in regulating the expression of pro-inflammatory mediators. Yu et al. found that miR-21 activates toll-like receptor 8 (TLR) resulting the level of TNFα and IL-12 increased in granulosa cells of PCOS (139). Furthermore, increased pro-inflammatory gene expression and induced NF-κB activation was observed in miR-146a^-/-^ mice (140). Another study demonstrated that CCL2 as the main target of miR-374a-5p, and also demonstrated that the transcription factor NF-κB can activate CCL2, suggesting that miR-374a-5p may play a role in regulating inflammatory responses (109). Li et al. described that miR-1224-5p exerts anti-inflammatory effects and alleviates PCOS by inhibiting the activation of the NF-κB signaling pathway (141).

**Figure 4:**
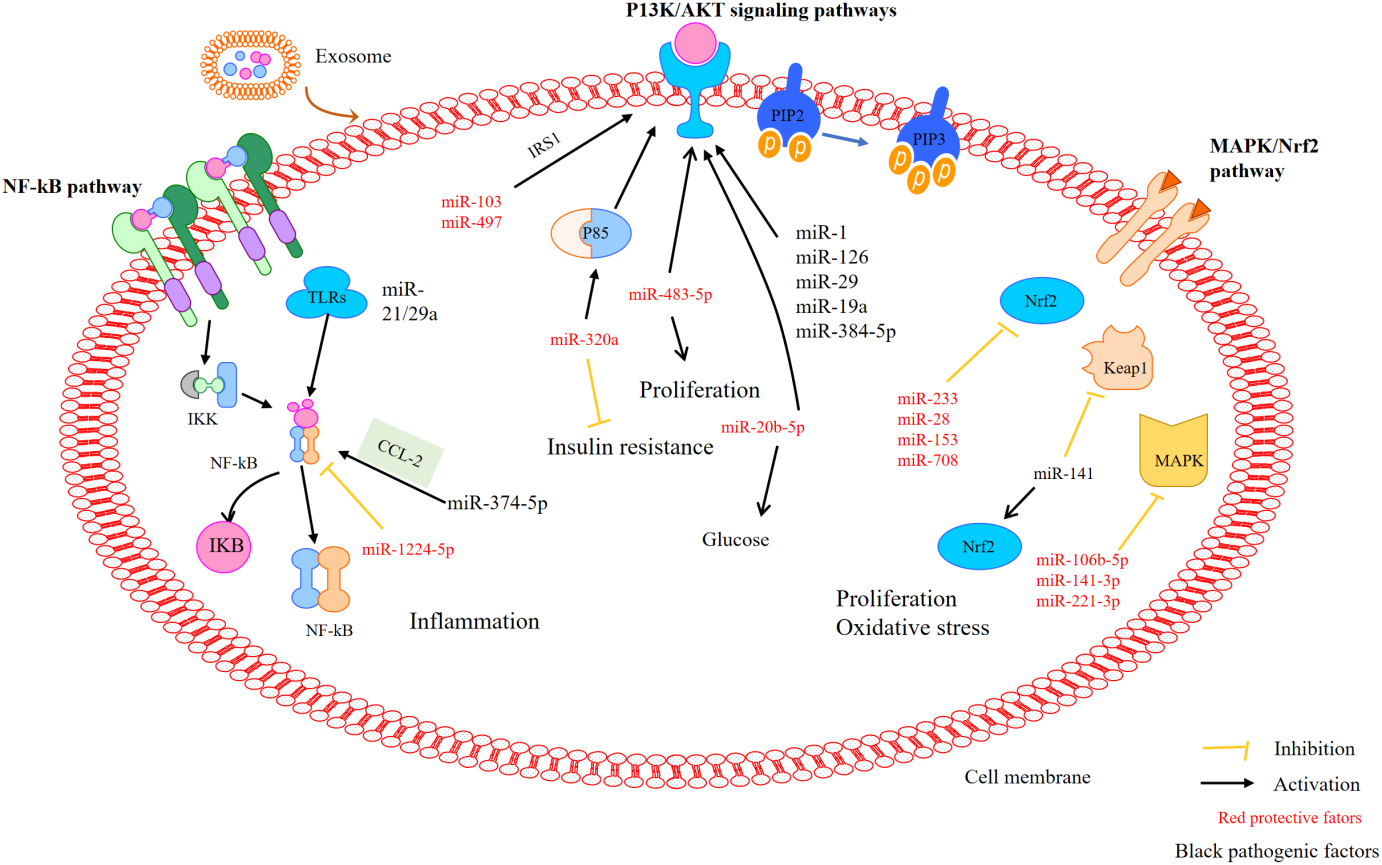
MiRNAs mediate pathways that may be involved in PCOS. MiRNAs mediate the activation of the P13K/Akt, NF-κB, and MAPK/Nrf2 signalling pathways in polycystic ovary syndrome.

Taken together, exosomes, especially those carrying miRNAs, are widely involved in the regulation of PCOS-related pathways such as PI3K/Akt, NF-κb, and AMPK/Nrf2 signaling pathways (**Figure 4**). This provides a new target for the treatment of PCOS. Unfortunately, there is not much research in this area, and the specific mechanism is still unclear, so further research is needed.

## 7 Conclusions and prospects

In recent years, research on exosomes has rapidly developed, covering almost all fields of physiology and pathophysiology. Exosomes are potential biomarkers and therapeutic agents for diseases. PCOS is a complex syndrome, involving diverse systems, primarily the reproductive and, endocrine systems. The paucity of a reliable diagnostic criteria and unclear pathogenesis pose as challenges in the development of therapeutics for PCOS. Abnormal miRNAs expression may be involved in the pathophysiology of PCOS, including reproductive functions, glucose metabolism and insulin sensitivity. However, existing research are primarily small-scale studies and the heterogeneity of their findings warrants further studies to elucidate their precise role in PCOS. Therefore, regulation of the circadian rhythm using miRNAs may be a novel therapeutic strategy for PCOS. In addition, an in-depth understanding of the interaction between genetics and the environment which lead to differential miRNAs expression may help elucidate the pathogenesis of polycystic ovarian diseases (79), which will provide new ideas for the prevention, diagnosis and treatment of PCOS.

## Author contributions

The envisaged role of all authors in the writing of the work is as follows: SL and QS: funding acquisition, project administration, supervision, validation, writing-review, and editing. WC: writing-original draft, writing-review, and editing. QH: supervision, validation, writing-review, and editing. ZW and XZ: writing-review and editing. All authors contributed to the article and approved the submitted version.

## Funding

This work was supported by the Science and Technology Bureau of Quanzhou (Grant number 2020CT003) and the Science and Technology project of the Fujian Provincial health commission (Grant number 2020CXB027).

## Acknowledgments

We are thankful to The Second Affiliated Hospital of Fujian Medical University for providing infrastructure facilities.

## Conflict of interest

The authors declare that the research was conducted in the absence of any commercial or financial relationships that could be construed as a potential conflict of interest.

## Publisher’s note

All claims expressed in this article are solely those of the authors and do not necessarily represent those of their affiliated organizations, or those of the publisher, the editors and the reviewers. Any product that may be evaluated in this article, or claim that may be made by its manufacturer, is not guaranteed or endorsed by the publisher.

## Abbreviations

ABCA1, ATP-binding cascade transporter A1; AKT, Protein kinase B; AMPK, AMP-activated protein kinase; AR, Androgen receptor ; BAML1, Brain and muscle arnt-like protein 1; BMI, Body mass index ; CAT, Catalase; CCL2, C-C motif chemokine ligand 2 ; CLOCK, Circadian locomotor output cycle kaput ; CR, Circadian rhythm; CREB, CAMP response element binding protein 1 ; CRP, C-reactive protein; CRTC2, CREB-regulated transcription coactivator 2; Cry, Cryptochrome; E2, Estradiol; ERK, Extracellular signal- regulated kinase; EVs, Extracellular vesicles; FSH, Follicle-stimulating hormone ; GABA, Gamma-aminobutyric acid; GCs, Granulosa cells ; GLUT4, Glucose transporter 4; GnRH, Gonadotropin-releasing hormone; GSIS, Glucose-activated insulin secretion; HA, Hyperandrogenism; HPG, Hypothalamic-pituitary-gonadal ; HPO, Hypothalamic-pituitary-ovarian ; HO-1, Heme oxygenase-1; IL1, Interleukin 1; IL6, Interleukin 6; IR, Insulin resistance; IRAK1, IL-1 receptor-related kinase 1; IRS, Insulin receptor substrate; Keap1, Kelch-like ECH-associated protein 1 ; KO, Knockout; LDL-C, Low-density lipoprotein cholesterol-C; LH, Luteinising hormone; Lhcgr, Luteinizing hormone receptor; MAPK, Mitogen- activated protein kinase; MCP-1, Monocyte chemotactic protein-1; MiRNA, MicroRNA; MRNA, Messenger RNA; MT, Melatonin; MV, Microbubbles; NAD, Nicotinamide adenine dinucleotide; NAMPT, Nicotinamide phosphoribosyl transferase; NcRNA, Non-coding RNA; NQO-1, phosphoamide adenine dinucleotide quinone oxidoreductase-1; Nrf2, Nuclear factor-erythrocyte 2-related factor 2; OS, Oxidative stress ; PCOS, Polycystic ovary syndrome; Per, Period; PI3K, Phosphatidylinositol 3 - kinase ; PPARγ, Peroxisome proliferator-activated receptor γ; ROR, Retinoic acid-related orphan receptor; RORE, ROR reaction element; ROS, Reactive oxygen species; SCN, suprachiasmatic nucleus; SIRT1, Protein deacetylase sirtuin 1; SOD, Antioxidant enzyme; TNFα, Tumor necrosis factorα; T2DM, Type 2 diabetes mellitus; VLDL-C, Very low-density lipoprotein cholesterol-C; WAT, White adipose tissue ; WHR, Waist-hip ratio

## Glossary

**Table:**

Abbreviation	Words
ABCA1	ATP-binding cascade transporter A1
AKT	Protein kinase B
AMPK	AMP-activated protein kinase
AR	Androgen receptor
BAML1	Brain and muscle arnt-like protein 1
BMI	Body mass index
CAT	Catalase
CCL2	C-C motif chemokine ligand 2
CLOCK	Circadian locomotor output cycle kaput
CR	Circadian rhythm
CREB	CAMP response element binding protein 1
CRP	C-reactive protein
CRTC2 Cry E2	CREB-regulated transcription coactivator 2 Cryptochrome Estradiol
ERK	Extracellular signal- regulated kinase
EVs	Extracellular vesicles
FSH	Follicle-stimulating hormone
GABA	Gamma-aminobutyric acid
GCs	Granulosa cells
GLUT4 GnRH	Glucose transporter 4 Gonadotropin-releasing hormone
GSIS	Glucose-activated insulin secretion
HA	Hyperandrogenism
HPG	Hypothalamic-pituitary-gonadal
HPO	Hypothalamic-pituitary-ovarian
HO-1	Heme oxygenase-1
IL1	Interleukin 1
IL6	Interleukin 6
IR	Insulin resistance
IRAK1	IL-1 receptor-related kinase 1
IRS	Insulin receptor substrate
Keap1	Kelch-like ECH-associated protein 1
KO	Knockout
LDL-C LH	Low-density lipoprotein cholesterol-C Luteinising hormone
Lhcgr	Luteinizing hormone receptor
MAPK	Mitogen- activated protein kinase
MCP-1	Monocyte chemotactic protein-1
MiRNA	MicroRNA
MRNA	Messenger RNA
MT	Melatonin
MV	Microbubbles
NAD	Nicotinamide adenine dinucleotide
NAMPT	Nicotinamide phosphoribosyl transferase
NcRNA	Non-coding RNA
NQO-1	phosphoamide adenine dinucleotide quinone oxidoreductase-1
Nrf2	Nuclear factor-erythrocyte 2-related factor 2
OS	Oxidative stress
PCOS Per	Polycystic ovary syndrome Period
PI3K	Phosphatidylinositol 3 - kinase
PPARγ	Peroxisome proliferator-activated receptor γ
ROR	Retinoic acid-related orphan receptor
RORE	ROR reaction element
ROS	Reactive oxygen species
SCN	suprachiasmatic nucleus
SIRT1	Protein deacetylase sirtuin 1
SOD TNFα	Antioxidant enzyme Tumor necrosis factorα
T2DM	Type 2 diabetes mellitus
VLDL-C	Very low-density lipoprotein cholesterol-C
WAT	White adipose tissue
WHR	Waist-hip ratio
